# Effectiveness and feasibility of short-course simulator training for robotic surgery novices – a randomized controlled trial (FastSim trial)

**DOI:** 10.1515/iss-2025-0003

**Published:** 2025-05-22

**Authors:** Sotirios Emmanouilidis, Kürsat Kirkgöz, Tina Groß, Benjamin Müssle, Anna Klimova, Daniel Stange, Thilo Welsch

**Affiliations:** Department of General, Visceral and Thoracic Surgery, Oberschwabenklinik Ravensburg, Akademisches Lehrkrankenhaus der Universität Ulm, Ravensburg, Germany; Department of General, Visceral and Thoracic Surgery, University Hospital Hamburg-Eppendorf, Hamburg, Germany; Department of General and Visceral Surgery, University of Ulm, Ulm, Germany; National Center for Tumour Diseases (NCT/UCC), Dresden, Germany; Core Unit for Data Management and Analytics, National Center for Tumour Diseases (NCT), Dresden, Germany; Department of Visceral, Thoracic and Vascular Surgery, Technische Universität Dresden, Dresden, Germany; Faculty of Medicine and University Hospital Carl Gustav Carus, Technische Universität Dresden, Dresden, Germany; Department of General, Visceral and Tumor Surgery, Krankenhaus Nordwest, Frankfurt, Germany

**Keywords:** robotic surgery, simulator training, surgical education, randomized controlled trial, training efficacy

## Abstract

**Objectives:**

As robot-assisted surgery (RAS) is on the rise, feasible and effective simulator training for surgical residents is needed. This study compares the effectiveness and feasibility of two compact simulator training protocols on the DaVinci skills simulator (dVSS) for preparing robotic surgery novices.

**Methods:**

In this randomized controlled trial, RAS novices were randomized 1:1 into a conventional group (Control), including four repetitions of five simulator exercises in two sessions, and a fast group (Fast), with two repetitions of the same five simulator exercises in a single training session. The primary endpoint was the mean efficiency score achieved in the final exercise.

**Results:**

Fifty-two participants (22 males) were randomized, 26 in each group. Forty-eight per cent were between 30 and 50 years old and most of the participants were residents (44.2 %) or consultants (42.3 %), 13.5 % were students. The primary endpoint results showed a mean efficiency score of 48.2 ± 26.7 (Fast) vs. 52.3 ± 30.4 (Control) in the intention-to-treat analysis (p=0.527). The most significant improvement in efficiency and penalty scores was observed between the 1st and 4th repetitions in the control group. Participants over 50 years old and consultants performed worse than younger participants, students and resident doctors. Participants interested in robotic surgery outperformed those with little or no interest.

**Conclusions:**

A training of 10–20 exercises on the dVSS with ascending levels is insufficient to succeed in complex simulation exercises but improves performance and motivation. These findings emphasize the need for tailored training programs and continuous skill development in robotic surgery.

## Introduction

The rise of robotic surgery has fundamentally changed prostatectomy procedures as well as elective abdominal surgery, including colorectal, gastric, pancreatic, liver, and esophageal resections and thoracic surgery [1], 2]. This trend is further fuelled by recent trials that have demonstrated improved short-term outcomes after minimally invasive robot-assisted resections [3], [4], [5]. In addition, the use of the robotic approach has significantly increased in emergency abdominal operations [6]. Currently, robotic abdominal surgery is even forecasted to become more common than laparoscopy for colorectal, pancreatic or esophageal resections by the year 2025 [7]. As a logical consequence, robotic surgery must be implemented in surgical training programs. However, surgical residents will have to learn robotic skills in addition to their other responsibilities within limited working hours.

Simulation training effectively enables specific robotic skills, prepares individuals for live procedures and is a key element of a structured modular robotic training curriculum [8], [9], [10]. Evidence of the effect of specific training programs is still rare, but single reports have demonstrated superior outcomes after robotic training and shortening of the learning curve for complex operations [11]. Although there is broad consensus on the core of the multimodal components of robotic training curricula, there is no clear definition of the optimal simulation exercises on the DaVinci skills simulator (dVSS) to reach a high proficiency level in a minimum amount of time. Therefore, the aim of the present trial was to evaluate and compare the feasibility and effectiveness of two defined, compact dVSS training protocols in preparing robotic surgery novices for the first steps during assisted clinical cases. Furthermore, personal characteristics and profiles, which enable fast or delayed adoption of robotic skills, were investigated.

## Methods

### Trial design

The study was designed as an investigator-initiated, randomized controlled single-center trial with two different parallel training designs (short [Fast]- and long-course [Control] sessions) on the dVSS (Intuitive Surgical, Inc., Sunnyvale, CA, USA). The reporting of the study methods and results was in line with the CONSORT statement [12]. Funding for the trial was covered by the institutional budget.

### Intervention and simulation exercises

**Figure 1:**
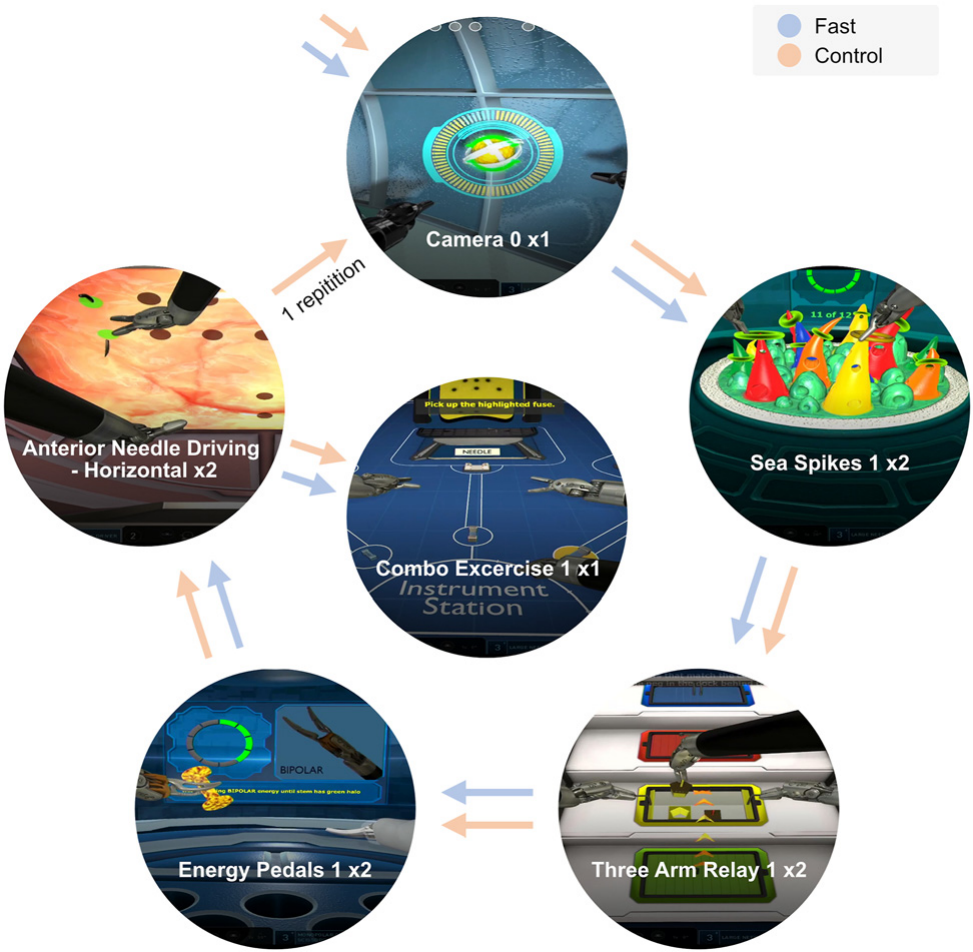
Training plan.

The participants in the intervention group performed fast training (Fast) on the dVSS with two repetitions of the same five simulator exercises in a single training session. The training in the control group (Control) comprised four repetitions of five simulator exercises in two sessions (on separate appointments) (Figure 1). The exercises were carefully designed to equip all participants with the basic skills required for a subsequent final exercise on the dVSS on the basis of the simulation content basic skill matrix (Table S1). The five training exercises were “Camera 0”, “Sea Spikes 1”, “Three Arm Relay 1”, “Energy Pedals 1”, and “Anterior Needle Driving – Horizontal” and were performed in this order. Each exercise was repeated once (two repetitions) except for the “Camera 0” exercise. After completion of the training, all participants accomplished an identical complex test exercise (“Combo Exercise”) once. Whereas the test exercise was subsequently scheduled after the training session in the Fast arm, the second training session was followed by the test in the Control group. Participants were allowed to implement breaks not exceeding 2 h between exercises. Since the study and the training sessions were conducted outside of regular operating hours, a compact schedule had to be implemented, and a 2.5-h time limit was set for the first training session. Participants exceeding this limit proceeded directly to the test exercise to avoid scheduling conflicts. In the Fast group, this had no effect in the analysis, but Control group participants were excluded from the protocol and analyzed “as treated”. A minimum time interval between the first and second training sessions for the control group of 24 h was mandatory.

### Outcomes

The primary end point of the study was the mean efficiency subtotal score (ESS) that was achieved in the final test exercise (“Combo Exercise”) by participants in the Fast or Control group. The ESS is a composite score provided by the dVSS and is calculated based on two performance parameters: (1) economy of motion and (2) time to complete. This endpoint was selected due to the importance of the economy of motion parameter, as emphasized by Walliczek–Dworschak et al. [13], who highlighted its significance as a key indicator of surgical efficiency. Additionally, the inclusion of the completion time parameter enables the evaluation of the impact of varying training durations on the time efficiency regarding completion of complex simulations.

The secondary end points included the overall (=ESS-penalty subtotal) and penalty scores in the final test; the individual learning curve based on the ESS and penalty scores in each exercise; the correlation of the ESS (in the training and test exercises) with the participants’ age, medical/surgical or laparoscopy experience; the evaluation of the duration and feasibility of the training sessions; and the participants’ subjective assessment of the importance of the robotic training.

### Participants, eligibility criteria and randomization

Eligible participants were physicians or medical students in their last year before the final exam (6th year medical students) aged≥18 years and had no experience performing operations on the DaVinci Si or Xi platform or the dVSS. Participants were also eligible if they had observed or assisted with robotic cases as first assistance. The exclusion criteria were that the participants would not be able to attend the appointments for the training sessions or that the time to complete the first training session exceeded 2.5 h. Potential participants were screened by the trial staff. If the participants fulfilled the inclusion criteria and consented to inclusion, they were randomized 1:1 using consecutively numbered, sealed opaque envelopes, and an appointment for the training sessions was scheduled. The randomization sequences were generated via the R software package (version 3.1.3, https://www.r-project.org) with fixed block sizes of 4. The randomization envelopes were prepared by an authorized trial coordinator at the center. The participants and the study coordinators were not blinded with respect to the intervention.

### Study visits and questionnaires

There were three study visits for the Control group and two for the Fast group, including the training and test sessions. Before the first training session, the participants were asked to complete a pretraining questionnaire, which covered basic participant characteristics such as age, gender, profession and medical experience (Table 1), as well as their individual perceptions of RAS, including their interest in RAS, experience, and perceived advantages of RAS (Table 2). After completing the final training session, the participants were further surveyed about their subjective evaluation of the final exercise (Table S2) as well as the entire training program (Table S3), and they were asked to reevaluate their perceptions of RAS (Table S4).

**Table 1:** Characteristics of the study participants.

Variable			Fast (n=26)	Control (n=26)	Total (n=52)	p-Value^a^
Gender	Female		16 (61.5)	14 (53.8)	30 (57.7)	0.779
	Male		10 (38.5)	12 (46.2)	22 (42.3)	0.779
Age	<30 y		8 (30.8)	6 (23.1)	14 (26.9)	0.755
	30–50 y		12 (46.2)	13 (50.0)	25 (48.1)	1.000
	>50 y		6 (23.1)	7 (26.9)	13 (25.0)	1.000
Dominant hand	Left-handed		1 (3.8)	2 (7.7)	3 (5.8)	1.000
	Right-handed		25 (96.2)	24 (92.3)	49 (94.2)	1.000
Professional position	Medical student		4 (15.4)	3 (11.5)	7 (13.5)	1.000
	Resident		11 (42.3)	11 (42.3)	22 (42.3)	1.000
	Consultant/Senior		11 (42.3)	12 (46.2)	23 (44.2)	1.000
Specialty	General surgery		10 (38.5)	12 (46.2)	22 (42.3)	0.779
	Gynecology		4 (15.4)	2 (7.7)	6 (11.5)	0.668
	Urology		1 (3.8)	4 (15.4)	5 (9.6)	0.350
	Vascular surgery		4 (15.4)	2 (7.7)	6 (11.5)	0.668
	Neurosurgery		2 (7.7)	2 (7.7)	4 (7.7)	1.000
	Internal medicine		0 (0.0)	1 (3.8)	1 (1.9)	1.000
	Medical student		4 (15.4)	3 (11.5)	7 (13.5)	0.419
Laparoscopic experience	None		14 (53.8)	12 (46.2)	26 (50.0)	0.782
	1-25/y		5 (19.2)	9 (34.6)	14 (26.9)	0.349
	>25/y		7 (26.9)	5 (19.2)	12 (23.1)	0.743
Technically proficient	Low		6 (23.1)	6 (23.1)	12 (23.1)	1.000
	Medium		18 (69.2)	18 (69.2)	36 (69.2)	1.000
	High		2 (7.7)	2 (7.7)	4 (7.7)	1.000
Experience with playing video or computer games	None		15 (57.7)	15 (57.7)	30 (57.7)	1.000
	Current and past (total)		11 (42.3)	11 (42.3)	22 (42.3)	1.000
	Current	≤10 h/wk	1 (3.8)	2 (7.7)	3 (5.8)	1.000
		>10 h/wk	0 (0.0)	0 (0.0)	0 (0.0)	–
	Past	≤10 h/wk	8 (30.8)	8 (30.8)	16 (30.8)	1.000
		<10 h/wk	2 (7.7)	1 (3.8)	3 (5.8)	1.000
Experience with playing musical instruments	None		7 (26.9)	15 (57.7)	22 (42.3)	0.048
	Beginner and advanced (total)		19 (73.1)	11 (42.3)	30 (57.7)	0.048
		Beginner	10 (38.5)	8 (30.8)	18 (34.6)	0.771
		Advanced	9 (34.6)	3 (11.5)	12 (23.1)	0.097

Values in parentheses are percentages unless indicated otherwise; y, years; h, hours; wk, weeks. ^a^Mann–Whitney-*U*-Test.

**Table 2:** Survey on robot-assisted surgery prior to training.

Variable		Fast (n=26)	Control (n=26)	Total (n=52)	p-Value^a^
Experience with robot-assisted surgery	None	22 (84.6)	20 (76.9)	42 (80.8)	0.726
	Introductory course	1 (3.8)	3 (11.5)	4 (7.7)	0.610
	Assistant	3 (11.5)	3 (11.5)	6 (11.5)	1.000
Interest in performing robot-assisted procedures	Does not apply	4 (15.4)	2 (7.7)	6 (11.5)	0.668
	Does rather not apply	8 (30.8)	4 (15.4)	12 (23.1)	0.324
	Partially applies	9 (34.6)	7 (26.9)	16 (30.8)	0.764
	Applies	5 (19.2)	13 (50.0)	18 (34.6)	0.040
Recommendation of robot-assisted procedures to patients	Unlikely	3 (11.5)	3 (11.5)	6 (11.5)	1.000
	Likely	23 (88.5)	23 (88.5)	46 (88.5)	1.000
Acceptance of novel medical technologies	Yes, even in developmental stage	17 (65.4)	10 (38.5)	27 (51.9)	0.095
	Yes, with proven benefits and cost advantages	9 (34.6)	14 (53.8)	23 (44.2)	0.264
	No/Skeptical	0 (0.0)	2 (7.7)	2 (3.8)	0.490
Conviction of benefits of robotic surgery	Does not apply	0 (0.0)	0 (0.0)	0 (0.0)	–
	Does rather not apply	3 (11.5)	4 (15.4)	7 (13.5)	1.000
	Partially applies	15 (57.7)	13 (50.0)	28 (53.8)	0.781
	Applies	8 (30.8)	9 (34.6)	17 (32.7)	1.000

Values in parentheses are percentages unless indicated otherwise. ^a^Mann–Whitney-*U*-Test.

### Sample size calculation and statistical analysis

For information about the score variability, we obtained a sample of scores from six participants during a training session with the conventional method before the trial started. The average score of the ESS was 62.2, with a standard deviation of ± 18.1 and a margin of error of 19.0. For the sample size calculation, we assumed that the score distributions of the training groups were independent and approximately normal, with a standard deviation of 18 points. We considered the difference in mean scores of 10 points, that is, approximately half of the observed margin of error, as being the clinically relevant minimum. Under these assumptions, a precision of ± 10 points in estimating the difference between mean scores with a 95 % confidence level can be reached with a sample size of 25 per group. To adjust for a drop-out, we planned to enroll one additional patient per group (n=26).

Descriptive variables are presented as medians and interquartile ranges (IQRs). The mean scores achieved in the groups were additionally described via 95 % confidence intervals (95 % CIs). Differences between the groups were analyzed via Fisher’s exact or *t*-test. To compare the survey results, a Likert scale was used, and each item score of the participants in a group was added and compared via the Mann‒Whitney U test. Subgroup analyses with a sample size of three or more were conducted with the Kruskal‒Wallis test, and repeated measurements were analyzed with Wilcoxon signed-rank and Friedman test. A p-value<0.05 was considered significant. The primary and secondary end points were analyzed as intention-to-treat (ITT) and per protocol (PP). Statistical calculations and plots were performed via the R software package (version 3.1.3).

## Results

**Figure 2:**
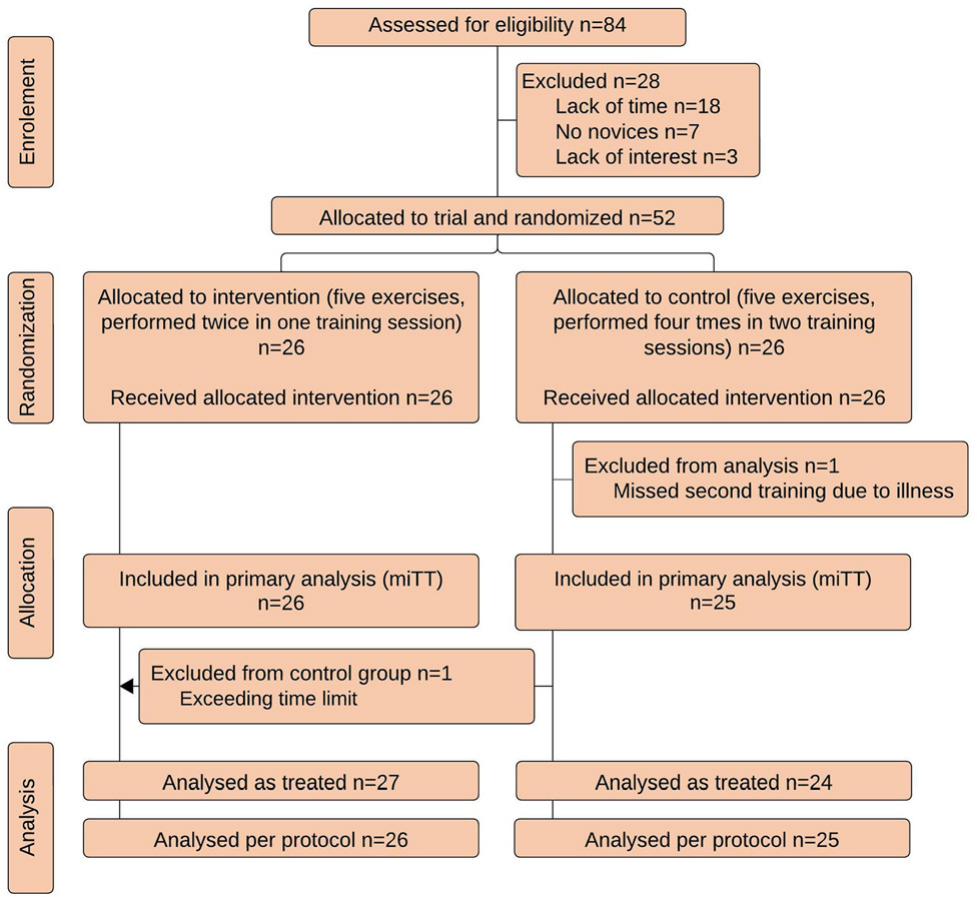
CONSORT flow diagram.

Between June and July 2023, 52 participants (22 males) were randomly assigned to the Fast (n=26) or Control (n=26) group (Figure 2). The majority of participants were aged between 30 and 50 years (48.1 %), and approximately one-quarter of participants were less than 30 or older than 50 years (Table 1). In total, 44.2 and 42.3 % of the participants were residents or consultants, respectively. Seven students were included (13.5 %). Almost all the physicians were trained in the field of surgery: 42.3 % were general/visceral surgeons, and 20 % were urologists or gynecologists. Half of the participants had prior laparoscopic experience, and 42.3 % were accustomed to video or computer gaming. There were no significant differences between the two groups, except that more participants in the intervention group played musical instruments (p=0.048).

With respect to robotic surgery experience, only three participants in each group had previously assisted in surgical cases (Table 2). However, one-third of the participants were motivated to learn about robot-assisted surgery, and almost 90 % would recommend the robotic approach to selected patients.

The primary endpoint (ESS) in the test exercise was not significantly different between the groups in either the intention-to-treat (ITT) or per protocol (PP) analysis: 48.15 ± 26.65 (Fast) vs. 52.28±30.42 (Control, ITT: p=0.527) and 48.19 ± 26.13 (Fast) vs. 52.42 ± 31.06 (Control, PP: p=0.473), respectively (Table 3). Similarly, the economy of motion, time to completion, penalty and overall scores were not significantly different. Repetitions of identical exercise were associated with improved performance, which was more pronounced in the control group (Figure S1). The most significant improvement in efficiency and penalty score was observed between the 1st and 4th repetitions in the Control group (Table S5).

**Table 3:** Primary and secondary endpoints of the final exercise.

	Fast (mean ± SD)	Control (mean ± SD)	p-Value^a^
Modified intention-to-treat population (n=51)	n=26	n=25	
Primary endpoint: Efficiency subtotal score	48.15 ± 26.65	52.28 ± 30.42	0.527
Per protocol population (n=51)	n=26	n=25	
Primary endpoint: Efficiency subtotal score	48.19 ± 26.13	52.42 ± 31.06	0.473
Secondary endpoints:			
Economy of motion score	27.28 ± 18.62	28.88 ± 16.48	0.880
Time to complete score	16.52 ± 18.74	20.48 ± 20.38	0.279
Penalty score	−54.73 ± 32.21	−54.96 ± 31.81	0.955
Overall score	19.19 ± 24.67	23.32 ± 27.07	0.696

SD, standard deviation. ^a^Mann*–*Whitney-*U*-Test.

**Figure 3:**
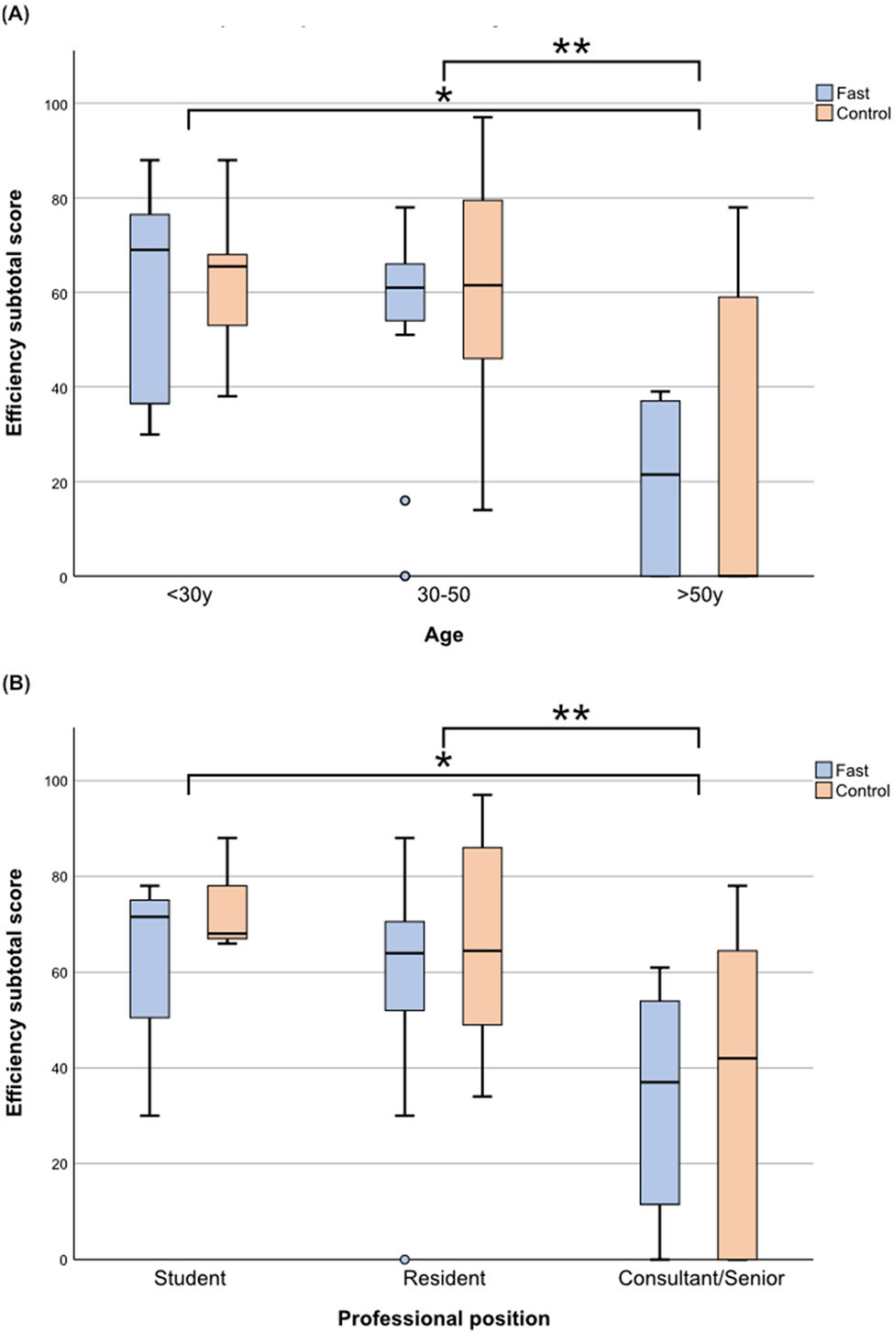
Performance stratified by age and experience according to subgroup. (A) Grouped boxplot of the efficiency subtotal scores in the final exercise stratified by age. (B) Grouped boxplot of the efficiency subtotal scores in the final exercise stratified by experience. *Defines a p-Value<0.05; **defines a p-Value<0.01. Abbreviation: y, years. Statistical testing was performed using the Kruskal*–*Wallis test with bonferroni correction.

**Figure 4:**
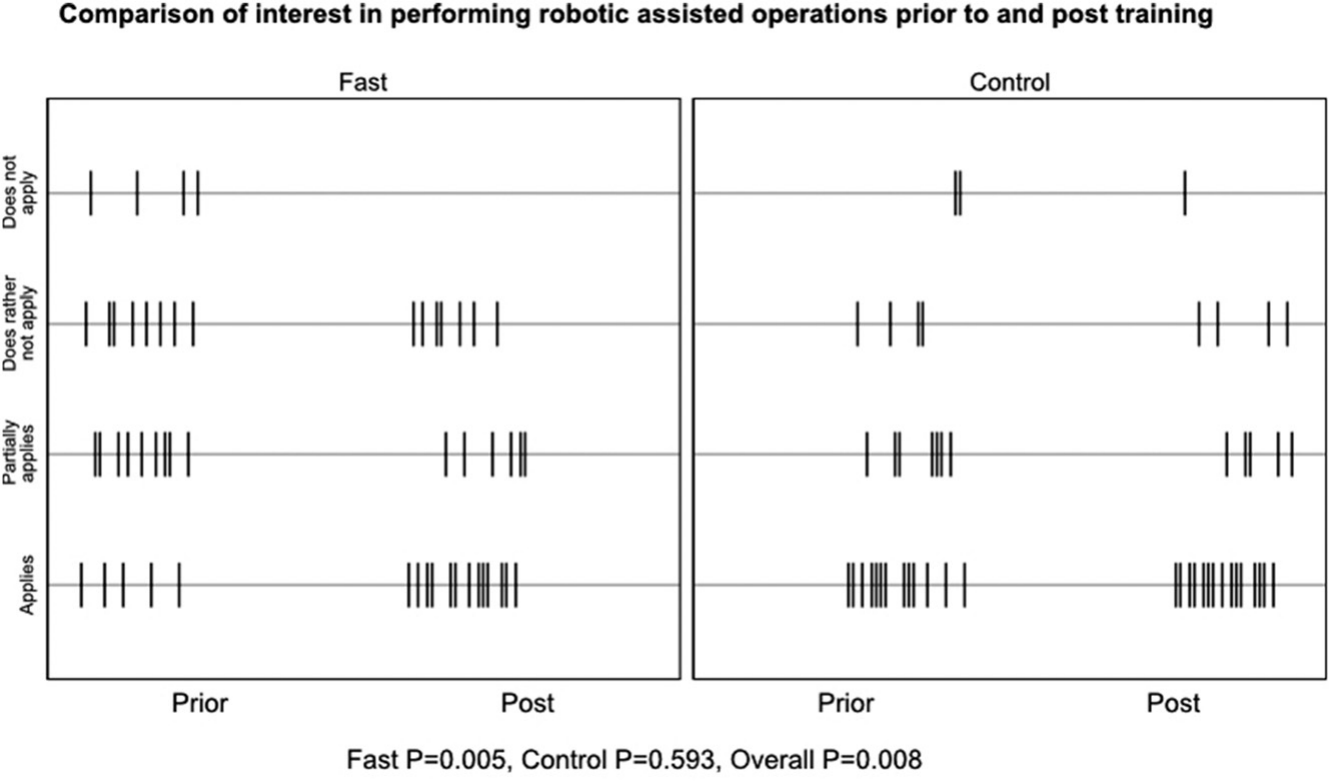
Comparison of interest in performing robotic-assisted operations prior to and after training. Statistical testing was performed using the Wilcoxon signed-rank test.

Participants over 50 years old and consultants performed worse than younger participants, students and resident doctors (Figure 3). Laparoscopic experience did not have a significant effect on performance (Figure S2). In addition, specialty, technical proficiency, experience with video or computer games, and playing musical instruments did not significantly impact the performance of the participants. A preexisting interest in RAS in the control group correlated with a significantly better test score in some exercises (Sea spikes 1; Three Arm Relay 1; Anterior Needle Driving – Horizontal; Table S6). All participants in the Fast group and 88 % of the participants in the Control group believed that more training would have yielded an improved performance test. Stress, concentration, and exhaustion levels were similar in both groups (Table S3). While 61 % of the Fast group and 36 % of the control group reported the training as physically strenuous, 50 and 56 %, respectively, found it mentally taxing. Nonetheless, neither physical nor mental effort significantly correlated with the overall scores achieved in the final exercise (p=0.099 and p=0.471, respectively, Kruskal‒Wallis test). The time to complete the training sessions was significantly shorter in the second training session (Control group, repetitions 3 and 4) and shorter than that in the training session in the Fast group. In contrast, the mean duration of the final test exercise was not significantly different (1,040.25 s [Control] vs. 1,119.73 s [Fast]) (Figure S3). The participants completed an entire training session in approximately 61 min (Fast group, without any interruption) and in approximately 92 min (Control group). The interest in performing robotic-assisted operations significantly increased after the training, especially in the Fast group (Figure 4).

## Discussion

This randomized trial focuses on the need for a simulator-based robotic training curriculum and is in line with recent studies that aimed to define optimal simulator exercises. One such study concluded that future trials should explore further limiting the duration of the training exercises to maintain the concentration and motivation of the trainees [13]. The participants in the latter study performed three different dVSS exercises with three repetitions each in two separate training sessions followed by two final text exercises (=18 training exercises). The present study considered 20 training exercises for the control group compared with 10 exercises for the intervention group and thus aimed to investigate a highly condensed training session. According to the primary endpoint analysis, there was no significant difference in the ESS score after these two training sessions. Both trainings resulted in an insufficient mean score of approximately 50 (max. 100), although a continuous increase in skill was observed during the repetitions. Both studies indicate that major increase in skill occurs between the 1st and 4th repetitions and that the expected ESS or overall score is dependent on the individual exercise.

In contrast to the findings of Walliczek–Dworschak et al., the present study revealed no significant difference in the subjective assessment of physical and mental effort or in the concentration levels between the fast and control groups. The physical and mental effort did not correlate with the final scores of the participants, suggesting that exhaustion from the training program is a minor factor in robotic training. However, compared with those in the control group, the daily and weekly working time, daily sleep time, and subjective fatigue in the fast group tended to correlate more strongly with test scores (not shown). This implies that a faster training program may make the test performance of participants more susceptible to fluctuations in their physical fitness. In support of this, studies on motor skill learning have shown that task repetition over different days enhances learning and consolidates motor skills, thus reducing performance fluctuations due to stress and fatigue [14].

Younger participants in both groups had significantly better test scores than older participants, corroborating previous studies that have found that younger age benefits robotic surgery training [15]. Similarly, students and residents outperformed consultants, likely due to the greater average age of the latter group. The advanced experience of consultants in minimally invasive and open surgery did not translate into better robotic surgery performance, which is consistent with findings showing that skills in these areas have limited transferability to robotic surgery [16]. A previous study revealed the benefits of recent gaming experience and previous video game experience on robotic surgery ability [17]. Thus, the high level of technological growth in our contemporary society, especially among young people and adolescents, is increasing their experience in computer skills and video games [18], which potentially serves as a beneficial factor for the acquisition of robotic surgery skills and might be an explanation for the better test results of younger participants.

Moreover, better test scores were observed in participants with a preexisting interest in robotic surgery. This aligns with previous studies that have shown that motivation can have a long-term impact on surgical skill performance, emphasizing the importance of motivation in surgical training [19]. Conversely, robotic surgical training could increase both interest in performing robotic-assisted operations and conviction regarding the advantages of robotic surgery. Consequently, including robotic surgery simulation training in medical school curricula appears to be a viable option. This inclusion could provide better insights into innovative surgical techniques and attract potential new surgeons.

Specifically for surgical residents, the results suggest that they may benefit most from simulator-based robotic training early in their education. The data also indicate that short, compact training – even if not sufficient for full proficiency – can still improve skills and motivation. This supports the feasibility of brief but structured simulator training in residency programs with limited time and resources. The impact of daily workload and sleep on performance in the Fast group should also be considered when planning curricula, favoring training during periods of lower clinical burden.

The present study has several limitations. The trial evaluated very short-course training sessions (maximum of 20 exercises), which resulted in an inefficient performance outcome to achieve a proficiency level with an ESS or overall score of >80. However, the trial highlighted several benefits of simulator training and provided insight into success and motivation. One may critically note that the trial design considered only one final test exercise after the simulator training, and it can be speculated that implementation of the text exercise before the training would have yielded more precise information. On the other hand, the authors held the opinion that the test exercise was too difficult without prior training.

In conclusion, training with 10–20 exercises on the dVSS with ascending levels is insufficient to succeed in complex simulation exercises but improves skills and motivates surgeons independent of their surgical experience. Experienced surgeons do not require less training than students or residents. The findings emphasize the need for tailored training programs and continuous skill development in robotic surgery for diverse participant profiles.

## Supplementary Material

Supplementary Material

## References

[1] Muaddi H, Hafid ME, Choi WJ, Lillie E, de Mestral C, Nathens A (2021). Clinical outcomes of robotic surgery compared to conventional surgical approaches (laparoscopic or open): a systematic overview of reviews. Ann Surg.

[2] Yeung TM, Larkins KM, Warrier SK, Heriot AG (2024). The rise of robotic colorectal surgery: better for patients and better for surgeons. J Robot Surg.

[3] Feng Q, Yuan W, Li T, Tang B, Jia B, Zhou Y (2022). Robotic versus laparoscopic surgery for middle and low rectal cancer (REAL): short-term outcomes of a multicentre randomised controlled trial. Lancet Gastroenterol Hepatol.

[4] Guerrini GP, Lauretta A, Belluco C, Olivieri M, Forlin M, Basso S (2017). Robotic versus laparoscopic distal pancreatectomy: an up-to-date meta-analysis. BMC Surg.

[5] Merboth F, Hasanovic J, Stange D, Distler M, Kaden S, Weitz J (2022). Change of strategy to minimally invasive esophagectomy—results at a certified center. Chirurg.

[6] Lunardi N, Abou-Zamzam A, Florecki KL, Chidambaram S, Shih IF, Kent AJ (2024). Robotic technology in emergency general surgery cases in the era of minimally invasive surgery. JAMA Surg.

[7] Ferrari D, Violante T, Novelli M, Starlinger PP, Smoot RL, Reisenauer JS (2024). The death of laparoscopy. Surg Endosc.

[8] Harji D, Houston F, Burke J, Griffiths B, Tilney H, Miskovic D (2023). The current status of robotic colorectal surgery training programmes. J Robot Surg.

[9] Puliatti S, Mazzone E, Dell’Oglio P (2020). Training in robot-assisted surgery. Curr Opin Urol.

[10] Sinha A, West A, Vasdev N, Sooriakumaran P, Rane A, Dasgupta P (2023). Current practises and the future of robotic surgical training. Surgeon.

[11] Jones LR, Zwart MJW, Molenaar IQ, Koerkamp BG, Hogg ME, Hilal MA (2020). Robotic pancreatoduodenectomy: patient selection, volume criteria, and training programs. Scand J Surg.

[12] Pandis N, Chung B, Scherer RW, Elbourne D, Altman DG (2017). CONSORT 2010 statement: extension checklist for reporting within person randomised trials. Br Med J.

[13] Walliczek-Dworschak U, Schmitt M, Dworschak P, Diogo I, Ecke A, Mandapathil M (2017). The effect of different training exercises on the performance outcome on the da Vinci Skills Simulator. Surg Endosc.

[14] Taraporewalla K, Barach P, van Zundert A (2024). Teaching medical procedural skills for performance. Clin Pract.

[15] Meier M, Horton K, John H (2016). Da Vinci© Skills SimulatorTM: is an early selection of talented console surgeons possible?. J Robot Surg.

[16] Kowalewski KF, Schmidt MW, Proctor T, Pohl M, Wennberg E, Karadza E (2018). Skills in minimally invasive and open surgery show limited transferability to robotic surgery: results from a prospective study. Surg Endosc.

[17] Kılınçarslan Ö, Türk Y, Vargör A, Özdemir M, Hassoy H, Makay Ö (2023). Video gaming improves robotic surgery simulator success: a multi-clinic study on robotic skills. J Robot Surg.

[18] Pop-Jordanova N (2024). Internet/video gaming: the relevance of a new phenomenon in the youth. Prilozi.

[19] Kannappan A, Yip DT, Lodhia NA, Morton J, Lau JN (2012). The effect of positive and negative verbal feedback on surgical skills performance and motivation. J Surg Educ.

