# Supplementary material for: Effectiveness and feasibility of short-course simulator training for robotic surgery novices – a randomized controlled trial (FastSim trial)
Source: Innov Surg Sci. 2025 May 22;11(2):115–22. doi: 10.1515/iss-2025-0003 (PMC13268609; doi:10.1515/iss-2025-0003)

Effectiveness and feasibility of short-course simulator training for robotic surgery novices—a randomized controlled trial (FastSim Trial)

Sotirios Emmanouilidis, Kürsat Kirkgöz, Tina Groß, Benjamin Müssle, Anna Klimova, Daniel Stange and Thilo Welsch

Supplementary material

Index

Supplementary Tables *page*

Table S1 Simulation Content Basic Skills Matrix 2
Table S2 Survey on final exercise (Combo Exercise) post training 2
Table S3 Survey on the training 3
Table S4 Survey on robot-assisted surgery post training 4
Table S5 Development of efficiency subtotal and penalty score (p-values) 4
Table S6 Efficiency subtotal score in exercises according to interest to robotic surgery 5

Supplementary Figures

Figure S1 Development of efficiency subtotal and penalty score 6
Figure S2 Comparison of efficiency subtotal scores in the final exercise in relation to laparoscopic experience 7
Figure S3 Comparison of training times among training exercises and final exercise 8

Supplementary Tables

Table S1 Simulation Content Basic Skills Matrix

	EndoWrist® Manipulation	Camera Clutching	Master/Finger Clutching	Energy Control	Fourth Arm Control	Needle Control and Driving
**Camera 0**		●	○			
**Sea Spikes 1**	●					
**Three Arm Relay 1**	○	○	○		●	
**Energy Pedals 1**				●		
**Anterior Needle Driving - Horizontal**						●
**Combo Exercise**	●	●	●	●	●	●

SKILLS FOCUS: ● = Primary ○= Secondary

Table S2 Survey on final exercise (Combo Exercise) post training

Variable		Fast (n=26)	Control (n=25)	Total (n=51)	P-value†
**The final exercise was easy**	Applies	1 (3.9)	2 (8)	3 (5.9)	1.000
	Partially applies	4 (15.4)	4 (16)	8 (15.7)	1.000
	Does rather not apply	14 (53.8)	11 (44)	25 (49)	.579
	Does not apply	7 (26.9)	8 (32)	15 (29.4)	.764
**I was well prepared for the final exercise**	Applies	8 (30.8)	3 (12)	11 (21.6)	.173
	Partially applies	14 (53.8)	14 (56)	28 (54.9)	1.000
	Does rather not apply	2 (7.7)	5 (20)	7 (13.7)	.248
	Does not apply	2 (7.7)	3 (12)	5 (9.8)	.668
**I am satisfied with my performance in the final exercise regardless of my preparation**	Applies	1 (3.9)	3 (12)	4 (7.8)	.290
	Partially applies	7 (26.9)	4 (16)	11 (21.6)	.499
	Does rather not apply	11 (42.3)	9 (36)	20 (39.2)	.776
	Does not apply	7 (26.9)	9 (36)	16 (31.4)	.555
**If I had prepared more, I could have achieved a better performance**	Applies	18 (69.2)	17 (68)	35 (68.6)	1.000
	Partially applies	8 (30.8)	5 (20)	13 (25.5)	.523
	Does rather not apply	0 (0)	2 (8)	2 (3.9)	1.000
	Does not apply	0 (0)	1 (4)	1 (2)	1.000

Values in parentheses are percentages unless indicated otherwise. †Mann-Whitney-U-Test.

Table S3 Survey on the training

Variable		Fast (n=26)	Control (n=25)	Total (n=51)	P-value†
**I found the training interesting**	Applies	26 (100)	24 (96)	50 (98)	.235
	Partially applies	0 (0)	1 (4)	1 (2)	.490
	Does rather not apply	0 (0)	0 (0)	0 (0)	-
	Does not apply	0 (0)	0 (0)	0 (0)	-
**I felt stressed during the training**	Applies	2 (7.7)	5 (20)	7 (13.7)	.248
	Partially applies	13 (50)	6 (24)	19 (37.3)	.083
	Does rather not apply	5 (19.2)	8 (32)	13 (25.5)	.349
	Does not apply	6 (23.1)	6 (24)	12 (23.5)	1.000
**I was able to concentrate well throughout the training**	Applies	15 (57.6)	12 (48)	27 (52.9)	.579
	Partially applies	9 (34.6)	10 (40)	19 (37.3)	.776
	Does rather not apply	1 (3.9)	3 (12)	4 (7.8)	.350
	Does not apply	1 (3.9)	0 (0)	1 (2)	1.000
**I was able to navigate well throughout the training**	Applies	8 (30.8)	10 (40)	18 (25.3)	.565
	Partially applies	11 (42.3)	12 (48)	23 (45.1)	.781
	Does rather not apply	7 (26.9)	3 (12)	10 (19.6)	.291
	Does not apply	0 (0)	0 (0)	0 (0)	-
**The training was physically demanding**	Applies	5 (19.2)	3 (12)	8 (15.7)	.703
	Partially applies	11 (42.3)	6 (24)	17 (33.3)	.237
	Does rather not apply	5 (19.2)	6 (24)	11 (21.6)	.743
	Does not apply	5 (19.2)	10 (40)	15 (29.4)	.132
**The training was mentally demanding**	Applies	4 (15.4)	5 (20)	9 (17.7)	.726
	Partially applies	9 (34.6)	9 (36)	18 (35.3)	1.000
	Does rather not apply	8 (30.8)	4 (16)	12 (23.5)	.324
	Does not apply	5 (19.2)	7 (28)	12 (23.5)	.523
**I found the training to be a good opportunity to learn about robotic surgery**	Applies	24 (92.3)	24 (96)	48 (94.1)	1.000
	Partially applies	2 (7.7)	0 (0)	2 (3.9)	.490
	Does rather not apply	0 (0)	1 (4)	1 (2)	.490
	Does not apply	0 (0)	0 (0)	0 (0)	-
**I would prefer to train with exercises closer to reality**	Applies	1 (3.9)	5 (20)	6 (11.8)	.099
	Partially applies	6 (23.1)	11 (44)	17 (33.3)	.144
	Does rather not apply	11 (42.3)	5 (20)	16 (31.4)	.132
	Does not apply	8 (30.8)	4 (16)	12 (23.5)	.324

Values in parentheses are percentages unless indicated otherwise. †Mann-Whitney-U-Test.

Table S4 Survey on robot-assisted surgery post training

Variable		Fast (n=26)	Control (n=25)	Total (n=51)	P-value†
**Conviction of benefits of robotic surgery**	Applies	13 (50)	13 (52)	26 (51)	1.000
	Partially applies	10 (38.5)	9 (36)	19 (37.3)	1.000
	Does rather not apply	2 (7.7)	2 (8)	4 (7.8)	1.000
	Does not apply	1 (3.9)	1 (4)	2 (3.9)	1.000
**Interest in performing robot-assisted procedures**	Applies	13 (50)	15 (60)	28 (54.9)	.577
	Partially applies	6 (23.1)	5 (20)	11 (21.6)	1.000
	Does rather not apply	7 (26.9)	4 (16)	11 (21.6)	.499
	Does not apply	0 (0)	1 (4)	1 (2)	.490
**I feel more positive about robot-assisted surgery after the training than before**	Applies	12 (46.1)	12 (48)	24 (47.1)	1.000
	Partially applies	10 (38.5)	7 (28)	17 (33.3)	.555
	Does rather not apply	2 (7.7)	4 (16)	6 (11.8)	.419
	Does not apply	2 (7.7)	2 (8)	4 (7.8)	1.000
**I will strive to gather more experience in robot-assisted surgery in the future**	Applies	15 (57.7)	15 (60)	30 (58.8)	1.000
	Partially applies	5 (19.2)	8 (32)	13 (25.5)	.349
	Does rather not apply	6 (23.1)	2 (8)	8 (15.7)	.248
	Does not apply	0 (0)	0 (0)	0 (0)	-

Values in parentheses are percentages unless indicated otherwise. †Mann-Whitney-U-Test.

Table S5 Development of efficiency subtotal and penalty score (p-values)

	Fast	Control					
**Efficiency subtotal score (P-values†)**	1 vs. 2	1 vs. 2	1 vs. 3	1 vs. 4	2 vs. 3	2 vs. 4	3 vs. 4
Sea spikes 1	**.003**	**.021**	**.000**	**.000**	1.000	.213	1.000
Three Arm Relay 1	**.025**	.051	**.001**	**.000**	1.000	**.020**	.438
Energy Pedals 1	**<.001**	**.000**	**.012**	**.000**	1.000	1.000	.239
Anterior Needle Driving - Horizontal	**<.001**	.237	**.001**	**.001**	.377	.437	1.000
**Penalty score (P-values†)**	1 vs. 2	1 vs. 2	1 vs. 3	1 vs. 4	2 vs. 3	2 vs. 4	3 vs. 4
Sea spikes 1	**.001**	.612	**.002**	**.000**	.283	**.021**	1.000
Three Arm Relay 1	**.048**	1.000	**.030**	**.014**	.086	**.043**	1.000
Energy Pedals 1	**.024**	**.005**	.826	**.022**	.357	1.000	.920
Anterior Needle Driving - Horizontal	.223	.377	**.011**	**.017**	1.000	1.000	1.000

†Wilcoxon Signed-Rank test for the Fast group and Friedman test with Bonferroni correction for the Control group.

Table S6 Efficiency subtotal score in exercises according to interest to robotic surgery

Exercise	Control			Fast		
	None/Rather no (Mean ± SD)	Yes/Rather yes (Mean ± SD)	P-value†	None/Rather no (Mean ± SD)	Yes/Rather yes (Mean ± SD)	P-value†
Camera 0	47.83 ± 31.42	41.60 ± 35.07	.855	39.83 ± 37.62	33.93 ± 34.32	.693
Sea spikes 1	45.95 ± 19.68	66.59 ± 17.18	**.024**	62.83 ± 12.97	57.18 ± 21,75	.456
Three Arm Relay 1	22.38 ± 29.95	50.52 ± 56.78	**.038**	37.33 ± 30.30	36.32 ± 28.63	.979
Energy pedals 1	79.47 ± 19.90	92.30 ± 5.52	.100	91.42 ± 9,68	86.00 ± 19.10	.605
Anterior Needle Driving - Horizontal	35.83 ± 28.53	69.91 ± 24.07	**.021**	58.75 ± 16.25	60.86 ± 22.94	.571
Combo Exercice	26.80 ± 36.70	58.65 ± 25.91	.095	42.67 ± 28.45	52.86 ± 25.10	.341

Abbreviations: SD, standard deviation. †Mann-Whitney-U-Test.

Supplementary Figures

Figure S1. Development of efficiency subtotal and penalty score in the different exercises (Camera 0, See Spikes 1, Three Arm Relay 1, Energy Peddals 1, Anterior Needle Driving). Numbers on the horizontal axis represent the consecutive number of exercise repetition. * defines a p-value <0.05; ** defines a p-value < 0.01; *** defines a p-value < 0.001. Statistical testing was performed using the Wilcoxon Signed-Rank test for the Fast group and the Friedman test with Bonferroni correction for the Control group.

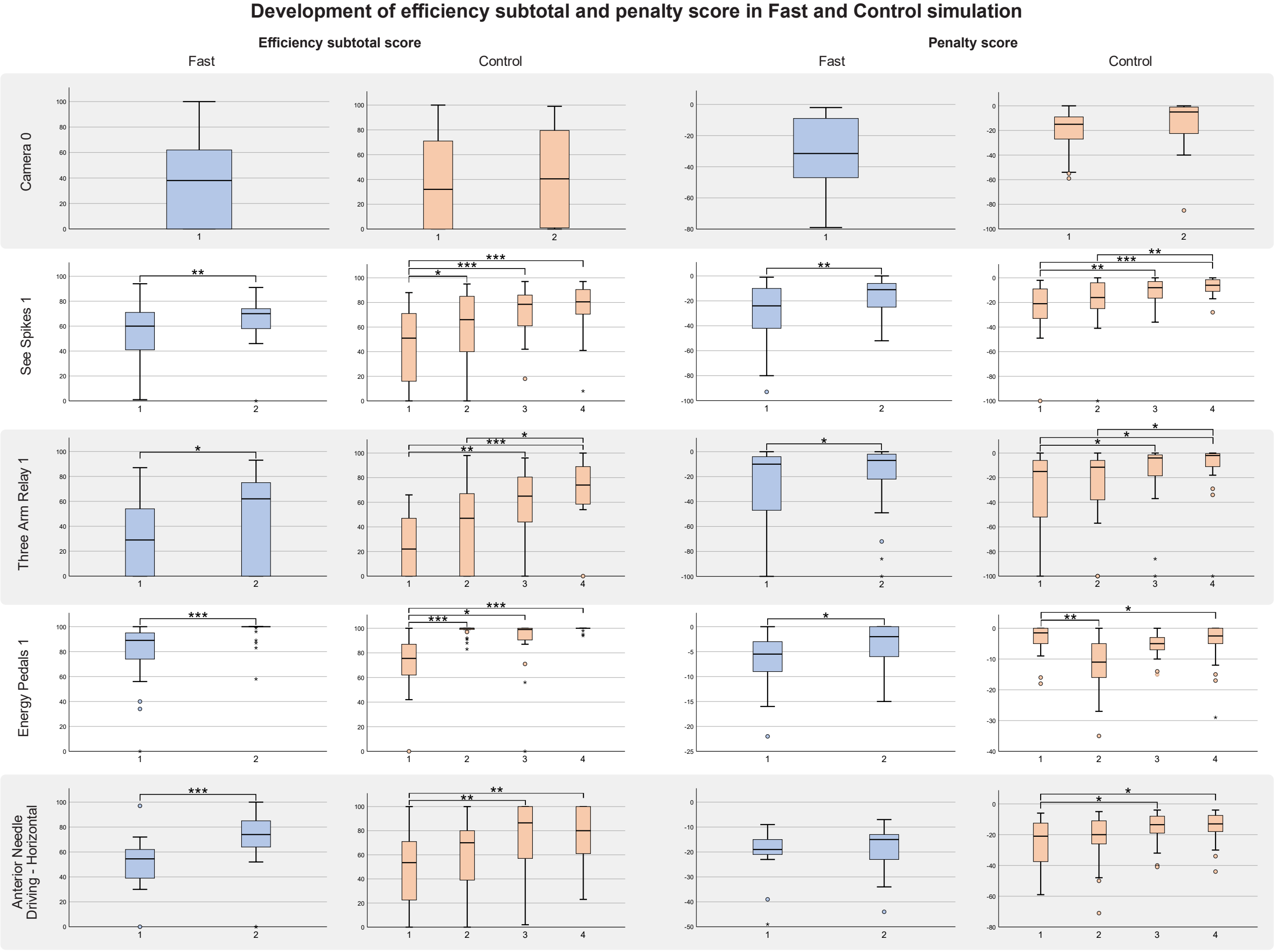

Figure S2. Comparison of efficiency subtotal scores in the final exercise in relation to laparoscopic experience
Abbreviations: y, years. Statistical testing was performed using Kruskal-Wallis test with Bonferroni correction.

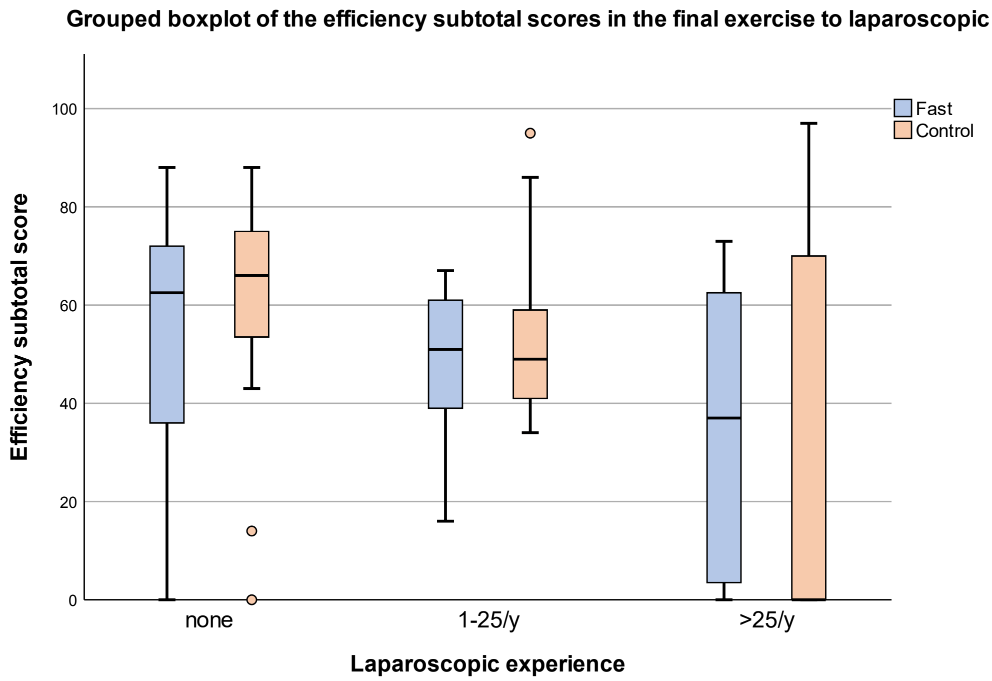

Figure S3. Comparison of training times among training exercises and final exercise (A) Grouped boxplot of training times among exercises. (B) Simple boxplot of training times in the final exercise. Statistical testing was performed using Kruskal-Wallis test with Bonferroni correction.

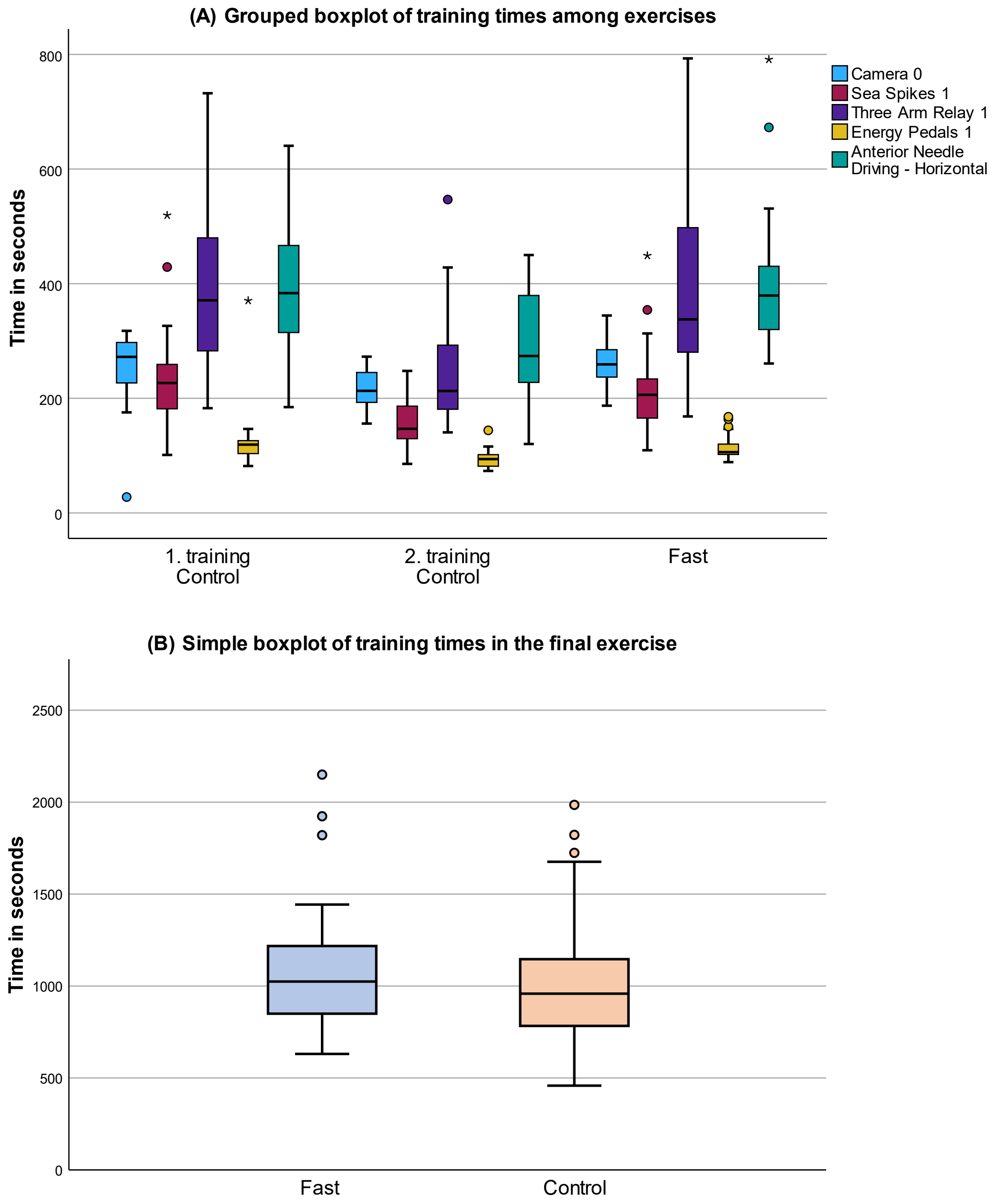

Supplement: Supplementary file 1 — Supplementary Material [file j_iss-2025-0003_suppl_001.pdf]
